# The Originally Established PBE Cell Line as a Reliable In Vitro Model for Investigating SIV Infection and Immunity

**DOI:** 10.3390/ijms26125764

**Published:** 2025-06-16

**Authors:** Xi-Chen Bai, Kohtaro Fukuyama, Leonardo Albarracin, Yoshiya Imamura, Fu Namai, Weichen Gong, Wakako Ikeda-Ohtsubo, Keita Nishiyama, Julio Villena, Haruki Kitazawa

**Affiliations:** 1 Laboratory of Animal Food Function, Graduate School of Agricultural Science, Tohoku University, Sendai 980-8572, Japan; bai.xichen.r4@dc.tohoku.ac.jp (X.-C.B.); kotaro.fukuyama.p8@dc.tohoku.ac.jp (K.F.); lalbarracin@herrera.unt.edu.ar (L.A.); yoshiya.imamura.p8@dc.tohoku.ac.jp (Y.I.); fu.namai.a3@tohoku.ac.jp (F.N.); gong.weichen.c1@tohoku.ac.jp (W.G.); wakako.ohtsubo.a7@tohoku.ac.jp (W.I.-O.); keita.nishiyama.a6@tohoku.ac.jp (K.N.); 2 Laboratory of Immunobiotechnology, Reference Centre for Lactobacilli (CERELA-CONICET), Tucuman 4000, Argentina; 3 Livestock Immunology Unit, International Education and Research Center for Food and Agricultural Immunology (CFAI), Graduate School of Agricultural Science, Tohoku University, Sendai 980-8572, Japan; 4 Division of Animal Immunology and Omics, International Education and Research Center for Food and Agricultural Immunology (CFAI), Graduate School of Agricultural Science, Tohoku University, Sendai 980-8572, Japan

**Keywords:** swine influenza virus, respiratory epithelial cells, innate immunity, porcine epithelial cells

## Abstract

Previously, we developed a porcine bronchial epithelial cell line designated as PBE cells and demonstrated that this cell line possesses functional Toll-like receptor 3 (TLR3), triggering the expressions of interferons (IFNs), antiviral factors, and inflammatory cytokines after its stimulation with the synthetic double-stranded ARN poly(I:C). In this work, we aimed to further characterize the PBE cell line as a reliable in vitro model for investigating swine influenza virus (SIV) infection and immunity. We evaluated the capacity of two SIV subtypes, H1N1 and H3N2, to replicate and induce cytopathic effects in PBE cells and to modulate the expressions of IFNs, antiviral factors, inflammatory cytokines, and negative regulators of the TLR signaling. We demonstrated that PBE cells are susceptible to both H1N1 and H3N2. SIV infected PBE cells inducing notable cytopathic effects as shown by the alteration of transepithelial electrical resistance (TEER) and cilia. Both SIV subtypes replicated in PBE cells in similar proportion and altered TEER values in comparable magnitudes. However, SIV H3N2 induced higher alterations of cilia than H1N1. SIV infection induced changes in all the immune factors evaluated in PBE cells. We detected quantitative differences when the subtypes H1N1 and H3N2 were compared. The fold expression changes of IFN-β, Mx1, Mx2, IFITM1, OAS1, OAS2, and OASL were higher in PBE cells infected with H3N2 than in cells challenged with H1N1. In addition, although both subtypes stimulated IL-8 expression, only the H3N2 induced IL-6 in infected PBE cells. SIV H1N1 and H3N2 also upregulated the expressions of the negative regulators A20, BCL-3, and MKP-1, while only H1N1 increased SIGIRR and Tollip. Immortalized respiratory cell lines from pigs can be useful in vitro systems for the study of viral infections and immune responses. These studies are of importance in the context of influenza infections not only for the agricultural field because pigs are natural hosts of these viruses but also because these animals serve as intermediate reservoirs of viruses that can threaten humans’ health. We demonstrated here that the PBE cell line can be a useful in vitro model to study SIV infection and immunity.

## 1. Introduction

Influenza virus A (IAV) is a major cause of respiratory disease not only in humans but also in pigs. Swine influenza virus (SIV) infection can induce fever, tachypnea, anormal breathing, and loss of appetite, affecting pigs’ growth. Although mortality associated to SIV is often very low, morbidity is high [1]. The study of SIV is of importance not only because of the economic impact related to the diminished weight gain in pigs [2], but also because these animals serve as intermediate reservoirs of viruses that can threaten humans’ health [3]. This threat is exemplified in the SIV that was capable of crossing from pigs to humans causing the influenza pandemic in 2009 [4].

Cell lines have enabled the advancement of our understanding of viral biology. In the case of IAV, the most commonly used cell lines are the African green monkey kidney epithelial cell line (Vero cells), the Madin–Darby canine kidney cell line (MDCK cells), and the baby hamster kidney cell line (BHK cells). However, these cells are derived from tissues that are not naturally infected by IAV, which limits the extrapolations that can be made about the infection of this virus in the respiratory tract in terms of its replication, cytopathic effect, and immune response. Furthermore, not all the cells of the respiratory epithelium are equally susceptible to IAV infection [5,6,7]. The impact of the human respiratory epithelium composition on IAV tropism and replication kinetics was evaluated in primary airway cultures. In these in vitro systems, it is possible to recapitulate the diverse epithelial cell-type composition. It was observed that several types of respiratory epithelial cells are susceptible to IAV infection; however, secretory and ciliated cells were predominantly affected [5]. Interestingly, ciliated cells allowed the highest virus production. This findings were confirmed in human airway organoids and ex vivo cultures of human bronchus explants, which showed that the IAV H1N1 subtype replicated in ciliated and secretory cells, but not basal cells [7]. The preferential tropism of IAV has been associated with the higher expression of the α-2,3-linked and α-2,6-linked sialic acid receptors in ciliated and secretory cells compared to other cell types in the human respiratory epithelium [8]. Of note, similar findings were described in pigs. Studies in precision-cut lung slices followed by culture and infection with SIV H3N2 showed that the virus infects the bronchiolar epithelium at a higher frequency than alveolar epithelial cells and that within the airways, both ciliated and secretory cells are more affected [6]. These studies show the need to develop in vitro systems that more accurately mimic the in vivo host environment to conduct more precise studies of IAV biology. This is particularly relevant for SIV, which has been less characterized than human IAV.

Primary cultures of porcine respiratory cells from the nasal turbinate, trachea, and lungs have been proposed to study SIV. It was shown that SIV H1N1 (swine IAV/MN08) replicated more efficiently in tracheal and lung primary cells while SIV H3N2 (swine IAV/IA07) replicated in the nasal turbinate [9]. Of note, while ciliated cells were detected in the initial cultures they gradually disappeared during subcultures. In line with these findings, it was reported that porcine nasal, tracheal, and bronchial epithelial cells cultured in an air–liquid interface system developed cilia and supported an efficient replication of SIV H3N2 (A/sw/NC/157671/2015 and A/sw/MO/A01476459/2012) [10]. Although useful, the use of primary porcine respiratory epithelial cell cultures has limitations. Primary cells are limited by the low number of passages before reaching senescence and altered gene expression [11], their frequent contamination with fibroblasts [12], as well as the donor variability [13]. To avoid these limitations, some porcine respiratory epithelial cell lines were developed, which have been shown to be useful to study SIV [12,14,15,16]. However, the immune response to SIV in those cell lines was not studied or was evaluated to a limited extent. Changes in the expressions of type I and III interferons (IFNs), as well as some interferon-stimulated genes and inflammatory cytokines, were described in porcine respiratory epithelial cell lines after SIV infection, but other important immune mediators like the negative regulators of the Toll-like receptors (TLRs) signaling pathway were not evaluated.

Previously, we developed a porcine respiratory epithelial cell line designated as PBE cells [17]. This cell line was established from the bronchial tissue of a neonatal pig. We observed that PBE cells can grow in vitro reaching confluence, establishing close contacts between the cells and developing cilia. The PBE cells express functional TLR3 as demonstrated by the fact that their stimulation with the synthetic double-stranded ARN poly(I:C) can trigger the expression of IFN-β, IFN-λ1, IFN-λ3, and the antiviral factors 2′,5′-oligoadenylate synthetase (OAS), interferon-induced GTP-binding Mx, protein kinase R (PKR), melanoma differentiation-associated protein 5 (MDA5), and retinoic acid-inducible gene I (RIG-I). The activation of the TLR3 pathway in PBE cells also induces the upregulation of tumor necrosis factor (TNF)-α, interleukin (IL)-6, IL-8, and monocyte chemoattractant protein-1 (MCP-1) as well as the differential modulation of negative regulators of the TLR signaling pathway. In this work, we aimed to further characterize the PBE cell line as a reliable in vitro model for investigating SIV infection and immunity. Therefore, we evaluated the capacity of two SIV subtypes, H1N1 and H3N2, to replicate and induce cytopathic effects in PBE cells and we evaluated the changes of immune factors that have been shown to play important roles in the outcomes of IAV infections in humans, pigs, and mice models including IFNs, antiviral factors, inflammatory cytokines, and negative regulators of the TLR signaling.

## 2. Results

### 2.1. SIV Efficiently Infects PBE Cells

**Figure 1:**
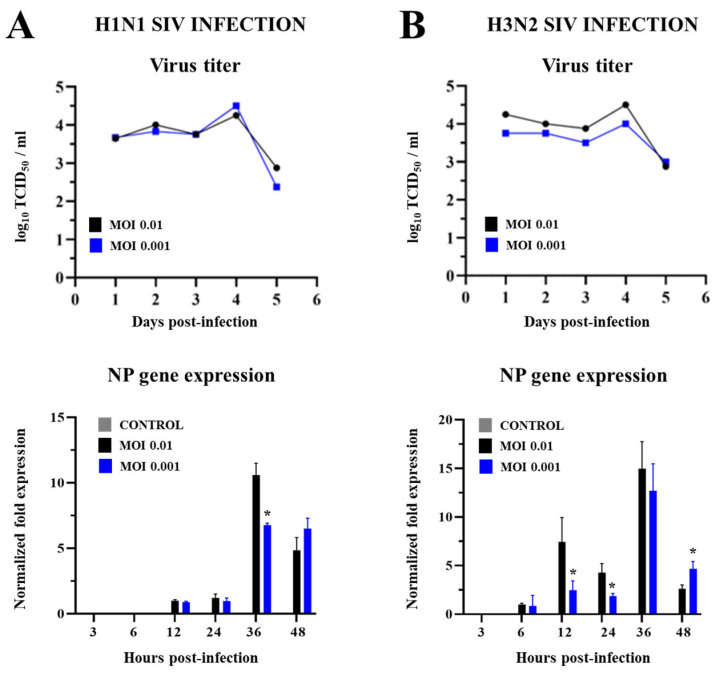
Viral titer and nucleoprotein (NP) expression in porcine bronchial epithelial (PBE) cells infected with swine influenza virus (SIV). The PBE cells were infected with SIV H1N1 (Tottori/41-6255/2018) (**A**) or SIV H3N2 (Kagoshima/37-7201/2019) (**B**) at a multiplicity of infection (MOI) of 0.01 or 0.001. The cell culture supernatants were harvested on days 1 to 5 post-infection to evaluate viral titers. Total RNA was extracted from PBE cells harvested at hours 3 to 48 post-infection for the determination of SIV NP expression. Data are presented as means ± SEM and results represent three independent experiments. Significant differences are shown compared to PBE cells infected with SIV MOI 0.01, at *p* < 0.05 (*).

We first aimed to evaluate whether SIV can infect PBE cells. For this purpose, we selected two subtypes of SIV: H1N1 and H3N2. PBE cells were challenged with these two subtypes of SIV at multiplicities of infection (MOIs) of 0.01 or 0.001, and the viral titers and the expression of the viral nucleoprotein (NP) were determined at several points after the infection (Figure 1).

It was observed that SIV H1N1 had a stable replication in PBE cells during the first three days after the challenge, with comparable viral titers (Figure 1A). The H1N1 titer increased significantly to a peak on the fourth day and decreased notably on the fifth day. No significant differences between the SIV H1N1 viral titers at the peak were detected for MOIs 0.01 and 0.001. Further analysis was conducted to evaluate the expression of the viral NP for H1N1 in PBE cells. As shown in Figure 1A, SIV H1N1 NP expression in PBE cells started to be detectable from 12 h post-infection, peaked at 36 h, and declined by 48 h. Of note, the NP expression was significantly higher for MOI 0.01 than MOI 0.001 only at h 36 post-infection.

Similarly, SIV H3N2 replicated steadily from the first to the third day and the viral titer significantly increased on the fourth day reaching a peak (Figure 1B). No significant differences between the SIV H3N2 viral titers at the peak were detected for MOIs 0.01 and 0.001. SIV H3N2 NP expression in PBE cells started to be detectable from 6 h post-infection, peaking at 36 h and declining at 48 h. Interestingly, the NP expression in PBE cells infected with MOI 0.01 was significantly lower at h 12 and 24, and higher at h 48, than cells infected with MOI 0.001 of SIV H3N2 (Figure 1B). These results demonstrate that PBE cells are permissive to both SIV H1N1 and H3N2.

**Figure 2:**
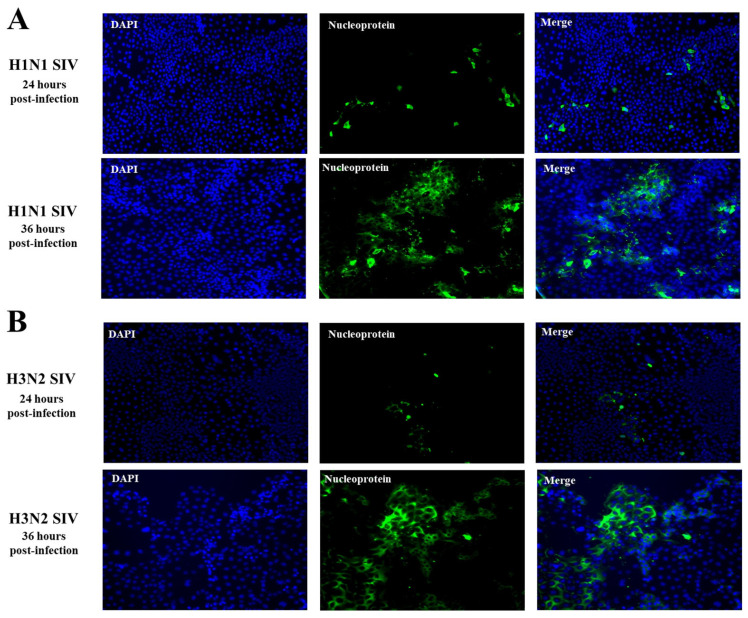
Viral nucleoprotein expression in porcine bronchial epithelial (PBE) cells infected with swine influenza virus (SIV). The PBE cells were infected with SIV H1N1 (Tottori/41-6255/2018) (**A**) or SIV H3N2 (Kagoshima/37-7201/2019) (**B**) at a multiplicity of infection (MOI) of 0.01. SIV nucleoprotein expression was evaluated in PBE cells at hours 24 and 36 post-infection by immunofluorescent staining. Blue: nucleus stained with DAPI, green: SIV nucleoprotein.

Consistent with the studies evaluating viral titers and NP expression, the immunofluorescence staining of SIV NP in challenged PBE cells revealed signs of viral replication at h 24 post-infection for both H1N1 (Figure 2A) and H3N2 (Figure 2B). At this point, small amounts of viral antigens were detected in PBE cells. However, when the cells were studied at h 36 post-infection a significant increase was noticed in SVI antigen, as shown by the larger and more extensive green-fluorescent signal (Figure 2). For both, H1N1 (Figure 2A) and H3N2 (Figure 2B) viral proteins accumulated around the cell nucleus at h 36.

**Figure 3:**
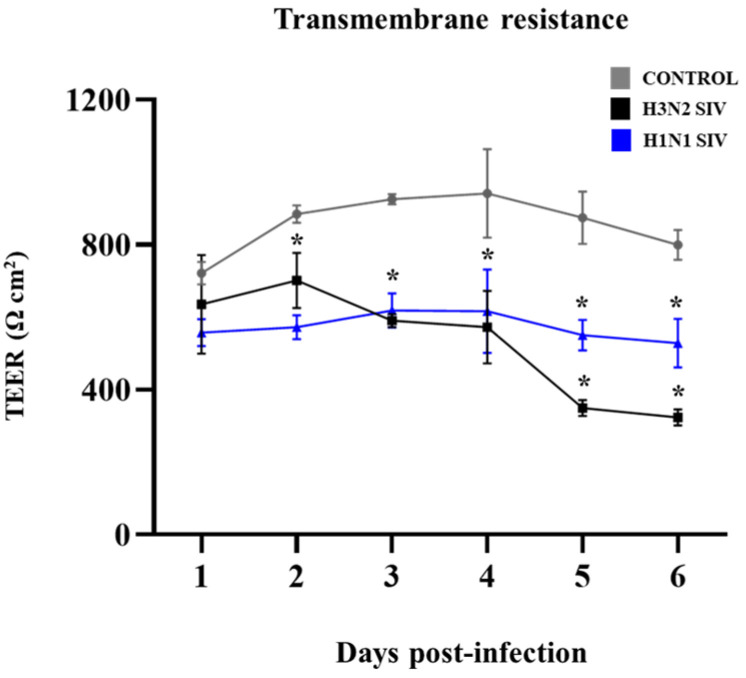
Transepithelial electrical resistance (TEER) in porcine bronchial epithelial (PBE) cells infected with swine influenza virus (SIV). The PBE cells were infected with SIV H1N1 (Tottori/41-6255/2018) or SIV H3N2 (Kagoshima/37-7201/2019) at a multiplicity of infection (MOI) of 0.01. TEER was measured on days 1 to 6 post-infection. Data are presented as means ± SEM and results represent three independent experiments. Significant differences are shown compared to non-infected PBE cells, at *p* < 0.05 (*).

We also evaluated the integrity of PBE cells’ monolayers after SIV infection by performing daily transepithelial electrical resistance (TEER) measurements during 6 days after the viral challenge (Figure 3). During the first three days, TEER increased in non-infected control PBE cells as they grew. In contrast, PBE cells infected with SIV H1N1 or H3N2 showed a lower increase in TEER during days 1 and 2 while the values of this parameter significantly decreased on day 3 post-infection compared to control cells. The drop in TEER values became more pronounced after day 4, with even greater differences being found between infected and control cells. Although the TEER values were similar on day 4 for both subtypes of SIV, the H3N2 SIV reduced TEER to levels significantly lower than H1N1 on days 5 and 6 (Figure 3). These results indicate that SIV infection disrupts the barrier integrity of PBE cells’ monolayers, H3N2 being the subtype with the higher ability to compromise the epithelial barrier integrity.

**Figure 4:**
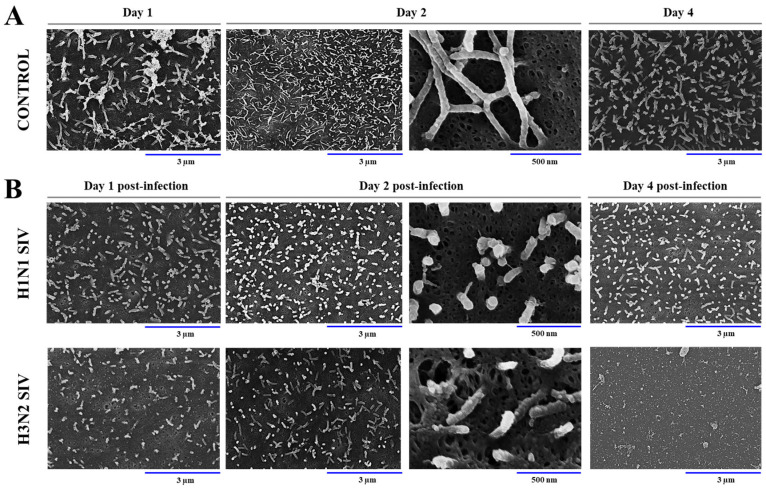
Scanning electron microscopy (SEM) analysis of porcine bronchial epithelial (PBE) cells infected with swine influenza virus (SIV). The PBE cells were infected with SIV H1N1 (Tottori/41-6255/2018) or SIV H3N2 (Kagoshima/37-7201/2019) at a multiplicity of infection (MOI) of 0.01. Non-infected control cells (**A**) and PBE cells challenged with SIV (**B**) were observed on days 1, 2, and 4.

Scanning electron microscopy was used to evaluate changes in the morphology of PBE cells after the infection with SIV. It was observed that non-infected PBE cells grow covering a large surface area and acquire a flattened shape. Of note, PBE cells had a surface layer of abundant cilia that grew in number and length from day 1 to 4 (Figure 4A).

When PBE cells were challenged with SIV, it was detected that cilia were notably shortened compared to the control group on days 1 and 2, for both H1N1 and H3N2 (Figure 4B). In addition, a reduction in the number of cilia was observed in PBE cells infected with SIV H3N2. By day 4, there was no further reduction in cilia length or number compared to day 1 in PBE cells infected with SIV H1N1. In contrast, cilia exhibited a shortening and almost completely disappeared in PBE cells infected with SIV H3N2 (Figure 4B). These findings suggest that SIV infection induces significant structural damage in PBE cells.

### 2.2. Innate Immune Response in PBE Cells Triggered by SIV Infection

**Figure 5:**
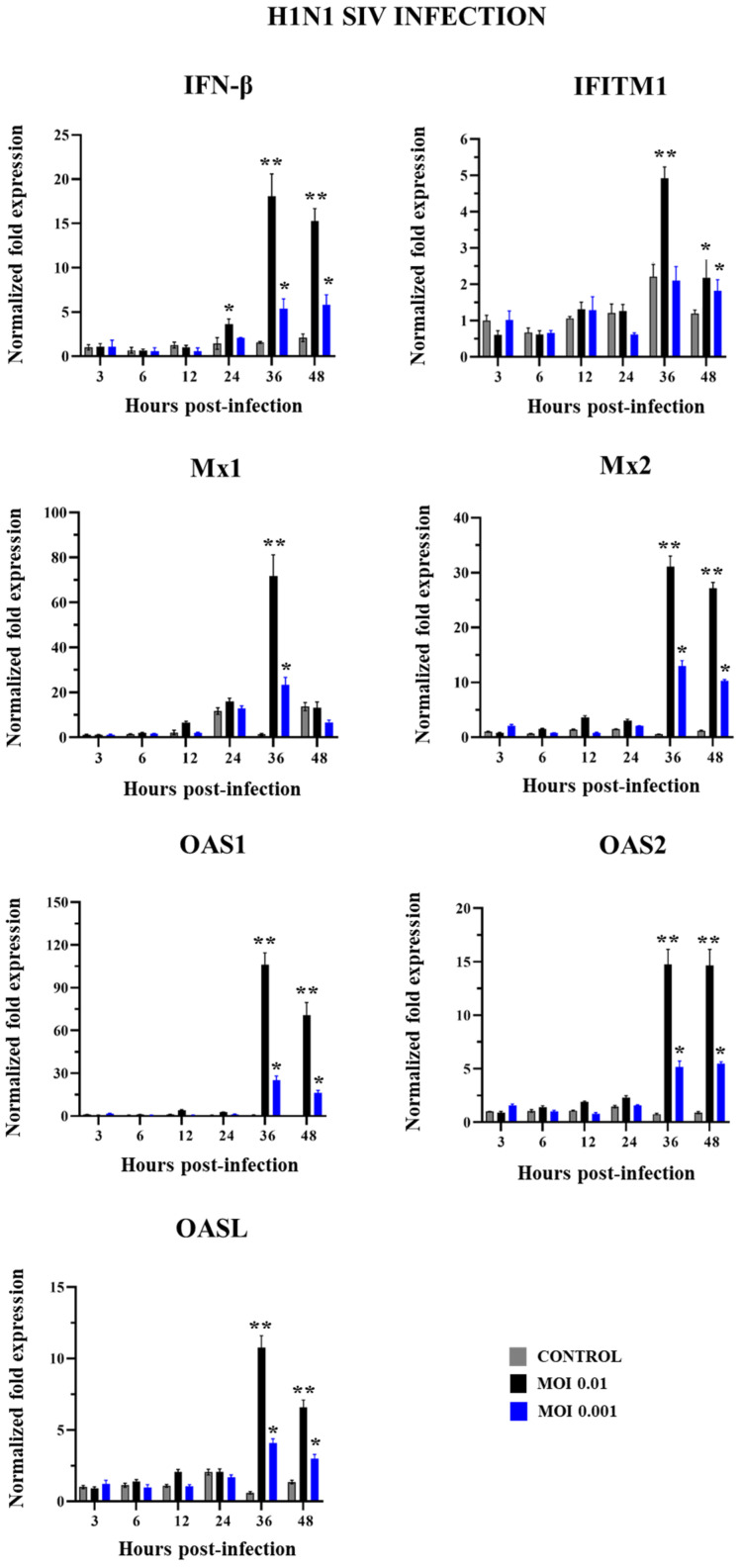
Expression of antiviral factors in porcine bronchial epithelial (PBE) cells infected with swine influenza virus (SIV). The PBE cells were infected with SIV H1N1 (Tottori/41-6255/2018) at a multiplicity of infection (MOI) of 0.01 or 0.001. The expressions of IFN-β, Mx1, Mx2, IFITM1, OAS1, OAS2, and OASL were determined by RT-PCR at hours 3 to 48 post-infection. Data are presented as means ± SEM and results represent three independent experiments. Significant differences are shown compared to non-infected PBE cells, at *p* < 0.05 (*) and *p* < 0.01 (**).

**Figure 6:**
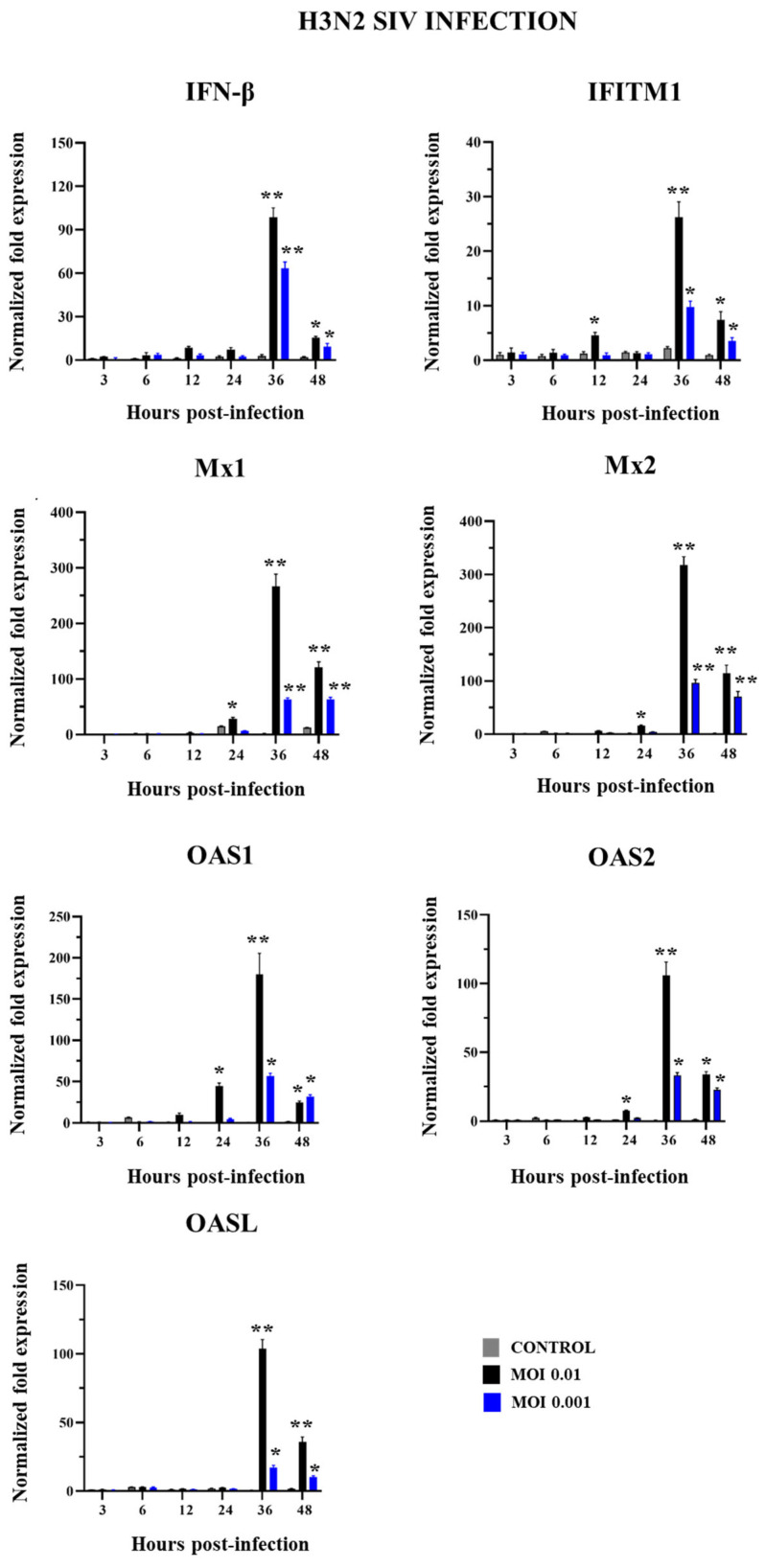
Expression of antiviral factors in porcine bronchial epithelial (PBE) cells infected with swine influenza virus (SIV). The PBE cells were infected with SIV H3N2 (Kagoshima/37-7201/2019) at a multiplicity of infection (MOI) of 0.01 or 0.001. The expressions of IFN-β, Mx1, Mx2, IFITM1, OAS1, OAS2, and OASL were determined by RT-PCR at hours 3 to 48 post-infection. Data are presented as means ± SEM and results represent three independent experiments. Significant differences are shown compared to non-infected PBE cells, at *p* < 0.05 (*) and *p* < 0.01 (**).

We next aimed to evaluate the antiviral innate immune response of PBE cells triggered by SIV infection. For this purpose, PBE cells were challenged with SIV H1N1 (Figure 5) or H3N2 (Figure 6) and the expression of IFN-β and the antiviral factors Mx1, Mx2, IFITM1, OAS1, OAS2, and OASL were evaluated at different hours post-infection. No significant differences were detected in the expression of all these factors within the first 24 h when non-infected control PBE cells were compared with cells challenged with SIV H1N1 (Figure 5). IFN-β and all the antiviral factors were augmented in infected PBE cells at h 36 and 48 compared to controls for both MOIs 0.01 and 0.001, although the expression levels of the immune factors were higher for the greater dose of SIV H1N1.

No significant differences were detected in the expression of IFN-β and the antiviral factors within the first 12 h when non-infected control PBE cells were compared with cells challenged with SIV H3N2 (Figure 6), except for IFITM1, which was increased in PEB cells challenged with MOI 0.01. This MOI of H3N2 also induced increases in the expressions of Mx1, Mx2, OAS1, and OAS2 at h 24. IFN-β and all the antiviral factors were significantly augmented in infected PBE cells at h 36 and 48 compared to controls for both MOIs 0.01 and 0.001, although the expression levels of the immune factors were higher for the greater dose of SIV H3N2.

The induction of antiviral factors expression was higher for H3N2 than H1N1 challenges. At h 36, the infection with SIV H1N1 (MOI 0.01) induced increases of approximately 20-, 70-, 30-, and 5-fold in IFN-β, Mx1, Mx2, and IFITM1, respectively, while these changes were 90-, 250-, 300-, and 25-fold for SIV H3N2 (MOI 0.01) (Figure 5 and Figure 6).

**Figure 7:**
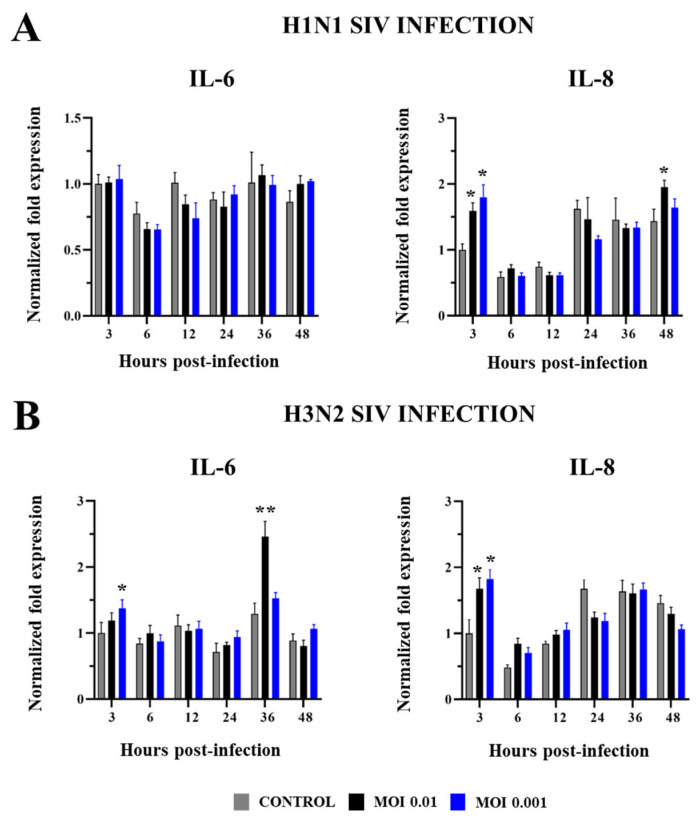
Expression of inflammatory cytokines in porcine bronchial epithelial (PBE) cells infected with swine influenza virus (SIV). The PBE cells were infected with SIV H1N1 (Tottori/41-6255/2018) (**A**) or SIV H3N2 (Kagoshima/37-7201/2019) (**B**) at a multiplicity of infection (MOI) of 0.01 or 0.001. The expressions of IL-6 and IL-8 were determined by RT-PCR at hours 3 to 48 post-infection. Data are presented as means ± SEM and results represent three independent experiments. Significant differences are shown compared to non-infected PBE cells, at *p* < 0.05 (*) and *p* < 0.01 (**).

We also measured changes in the expressions of the inflammatory cytokines IL-6 and IL-8 in PBE cells infected with SIV (Figure 7). No significant differences were detected in IL-6 expression when non-infected control PBE cells and cells challenged with SIV H1N1 MOIs 0.01 or 0.001 were compared (Figure 7A). In contrast, SIV H3N2 at MOIs 0.001 and 0.01 increased IL-6 expressions at h 3 and 36, respectively (Figure 7B). Both subtypes of SIV augmented the expression of IL-8 in PBE cells compared to controls early at h 3, and with no significant differences between MOIs 0.01 and 0.001. In addition, increases in IL-8 compared to controls were detected in PBE cells challenged with H1N1 MOI 0.01 at h 48 post-infection (Figure 7A). The magnitude of the changes induced by H1N1 and H3N2 in IL-8 expression was similar since the fold changes were approximately two in both cases.

**Figure 8:**
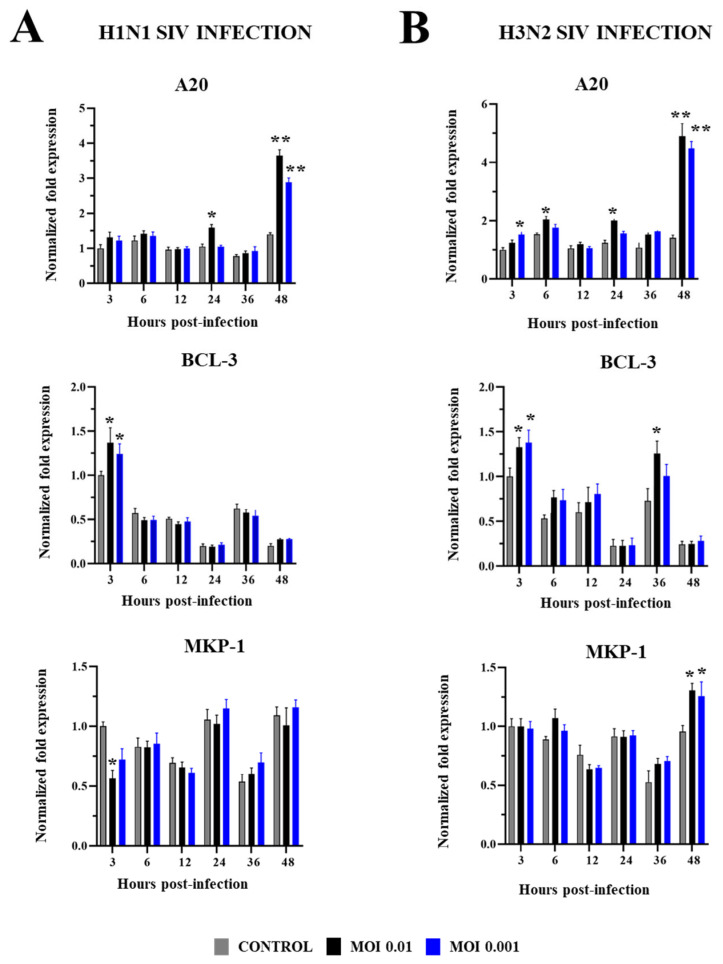
Expression of the negative regulators A20, BCL-3, and MKP-1 in porcine bronchial epithelial (PBE) cells infected with swine influenza virus (SIV). The PBE cells were infected with SIV H1N1 (Tottori/41-6255/2018) (**A**) or SIV H3N2 (Kagoshima/37-7201/2019) (**B**) at a multiplicity of infection (MOI) of 0.01 or 0.001. The expressions of the negative regulators of the TLR signaling pathway were determined by RT-PCR at hours 3 to 48 post-infection. Data are presented as means ± SEM and results represent three independent experiments. Significant differences are shown compared to non-infected PBE cells, at *p* < 0.05 (*) and *p* < 0.01 (**).

**Figure 9:**
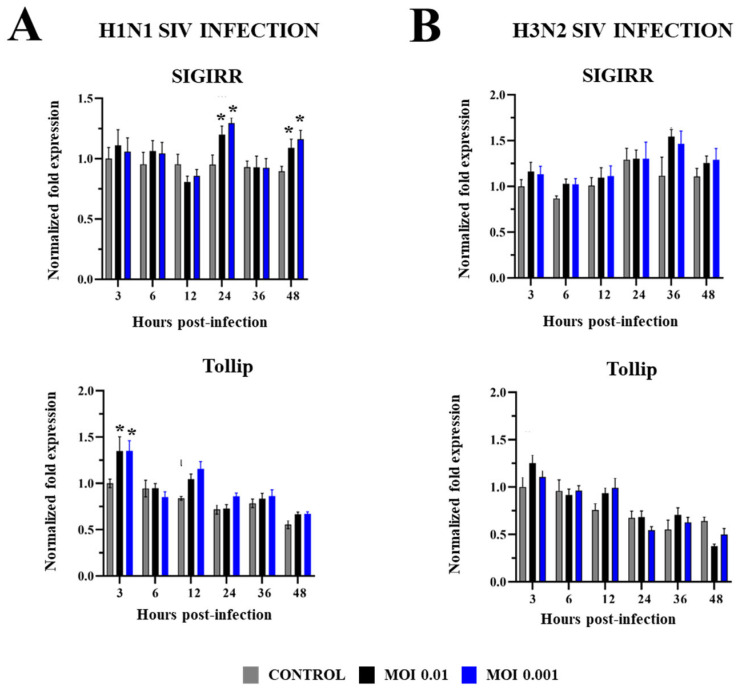
Expression of the negative regulators SIGIRR and Tollip in porcine bronchial epithelial (PBE) cells infected with swine influenza virus (SIV). The PBE cells were infected with SIV H1N1 (Tottori/41-6255/2018) (**A**) or SIV H3N2 (Kagoshima/37-7201/2019) (**B**) at a multiplicity of infection (MOI) of 0.01 or 0.001. The expressions of the negative regulators of the TLR signaling pathway were determined by RT-PCR at hours 3 to 48 post-infection. Data are presented as means ± SEM and results represent three independent experiments. Significant differences are shown compared to non-infected PBE cells, at *p* < 0.05 (*).

Finally, in PBE cells infected with SIV we evaluated changes in the expression of negative regulators of the TLR signaling: B-cell chronic leukemia protein 3 (BCL-3), ubiquitin-editing protein A20, mitogen-activated protein kinase 1 (MKP-1), single immunoglobulin interleukin-1-related receptor (SIGIRR), and the Toll-interacting protein (Tollip) (Figure 8 and Figure 9). H1N1 and H3N2 induced increases in the expressions of A20 and BCL-3, for both MOIs 0.01 and 0.001. The upregulation of BCL-3 was observed early at h 3 post-infection while the most remarkable change in A20 was detected at h 48 for both subtypes of SIV. No differences were detected in the expression of these negative regulators when the two MOIs were compared. A20 was also upregulated at h 24 by H1N1 MOI 0.01 while H3N2 MOI 0.01 increased BCL-3 at h 36 and A20 at h 6 and 24 (Figure 8). The magnitude of the changes induced by H1N1 and H3N2 in BCL-3 expression (h 3) was similar since the fold changes were approximately 1.6 in both cases. In addition, A20 (h 48) increased approximately 3.5- and 4.5-fold in H1N1 and H3N2 infections, respectively. Interestingly, MKP-1 expression was reduced in PBE cells infected with SIV H1N1 MOI 0.01 while the two doses of SIV H3N2 increased MKP-1 at h 48 (Figure 8). Only SIV H1N1 induced increments in the expressions of SIGIRR and Tollip. SIGIRR was upregulated at h 24 and 48 while Tollip was enhanced at h 3. No significant differences were detected when MOIs 0.01 and 0.001 were compared (Figure 9).

## 3. Discussion

Cell lines of human respiratory epithelial origin were successfully used to investigate virus–host interactions (reviewed in [13]). However, primary cultures or cell lines of porcine respiratory epithelial origin have been studied only to a limited extent. Primary porcine respiratory epithelial cell cultures have limitations since these cells can reach senescence and altered gene expression after a low number of passages [11], are often contaminated with fibroblasts [12], and can respond to viral infections in a variable way depending on the donor [13]. To avoid these limitations, some porcine respiratory epithelial cell lines were developed, which have been shown to be useful to study SIV. The MK1-OSU cell line was originally established from the distal trachea of a piglet [14]. It was shown that both H1N1 and H3N2 SIV replicated efficiently in this cell line with peaks between h 24 and 36 post-infection. Similarly, the NPTr cell line was derived from the trachea of a newborn pig and was shown to allow the replication of human, swine, and avian IAV [15]. In NPTr cells infected with H3N2 SIV (strain A/Swine/Bissendorf/IDT1864/2003), viral RNA was detected at 1 h post-infection and reached its highest levels at h 24 [18]. The siNEC and siTEC cell lines were established from porcine nasal and tracheal respiratory epithelial cells, respectively, and were shown to support the replication of both human and porcine IAV [12]. In these cell lines, the peaks of SIV titers were detected between h 48 and 72 post-infection. Of note, although both cell lines can form tight junctions, only siTEC cells are ciliated, and consequently, the siNEC cells did not support replication of SIV H3N2 (A/swine/Iowa/13-1015/2010) as effectively as the siTEC cells. On the other hand, the hTERT PBE cell line, originated from a piglet’s bronchial epithelial cells, was shown to be susceptible to SIV H3N2, although viral infection was evaluated only at one point after the challenge [16]. In all these studies the replication of SIV induced cytopathic effects including scattered growth, cell rounding, cilia alterations, and reduction of TEER values.

Previously, we developed the PBE cell line that originated from the bronchial epithelium of a neonatal pig [17]. We showed that these cells can grow reaching confluence and expressing cilia. We also demonstrated that PBE cells possess a functional TLR3 suggesting that this cell line could be a useful in vitro tool to study viral infections that affect the porcine respiratory epithelium. In this work, we further advanced in the characterization of this cell line as an in vitro model by demonstrating that PBE cells are susceptible to SIV infection, allowing the replication of two subtypes: H1N1 and H3N2. In our hands, the challenge of PBE cells with SIV allowed the replication of the viruses with peaks at day 4 post-infection, and with notable cytopathic effects, as shown by the alteration of TEER and cilia. Of note, both SIV subtypes replicated in PBE cells in similar proportion and altered TEER values in comparable magnitudes. However, SIV H3N2 induced higher alterations of cilia than H1N1. Modification of cilia function and development has been demonstrated in the respiratory epithelium after IAV infection. It was reported that the epithelial cell layer becomes thinner after IAV challenge due to the loss of ciliated cells through apoptosis [19]. This alteration of the respiratory epithelium has been corroborated in vitro as well as in pathological studies of samples from IAV-infected airways in humans [20,21]. Similar findings were described for SIV infection. Studies performed in precision-cut lung slices, in which the porcine respiratory epithelium was exposed to SIV H3N2 (A/sw/Bissendorf/IDT1864/2003), showed that after 48 h post-infection there was a decrease in ciliary activity [6]. Of note, cilia alterations appeared to depend on the amounts of viruses generated during infection. Interestingly, avian IAV that replicated less efficiently induced only partial cilia modifications. Using in vivo challenge experiments in pigs, it was reported that H1N1 2009 IAV strains isolated in 2009–2010 were significantly more virulent than the strains from 2014–2015 [22]. The in vitro studies with air–liquid interface airway epithelial cell cultures showed that the viruses from 2009–2010 replicated, reduced thickness of the epithelial cell layer, and induced loss of ciliated cells in higher proportions than viruses from 2014–2015. PBE cells then respond to SIV infection, as does the respiratory epithelium, and can therefore be a valuable tool to investigate the biology of viral infection as well as alternatives to reduce the cytopathic effects.

We also observed here that the challenge of PBE cells with SIV induced a complex innate immune response characterized by changes in the expressions of type I IFNs, antiviral factors, inflammatory cytokines, and negative regulators of the TLR signaling pathway. We detected quantitative differences in the immune factors when the subtypes H1N1 and H3N2 were compared. The fold expression changes of IFN-β, Mx1, Mx2, IFITM1, OAS1, OAS2, and OASL were higher in PBE cells infected with H3N2 than in cells challenged with H1N1. In addition, although both subtypes stimulated IL-8 expression, only the H3N2 induced IL-6 in infected PBE cells. These immune factors have been shown to participate in the defense against IAV in human cells and animal models. Several studies have shown that respiratory epithelial cells can produce various host restriction factors in response to IAV infection [23]. The most studied and well characterized are type I IFNs, which play a central role in virus–host interactions [24]. The IFN-β produced by epithelial cells in response to IAV can induce the expression of antiviral factors in the same and in surrounding cells. Several antiviral factors contribute to IAV elimination including Mx, OAS, and IFITM proteins. Mx GTPases have the capacity to inhibit viruses during the early stages of the replication, including IAV [25]. In this regard, it was shown that the disruption of the Mx1 gene can lead to a severe loss of protective innate immunity against IAV in mice, resulting in severe infection and rapid death [26]. On the other hand, OAS triggers the dimerization and activation of RNase L, which then cleaves viral RNA and limits replication [27]. Studies comparing the IAV infection in wild-type and *Oasl1*^−/−^ mice showed that the latter group have significantly higher airway hyperreactivity characterized by more severe peribronchiolar infiltration of inflammatory cells [28]. Single-cell RNAseq studies performed with cultures of human tracheal–bronchial epithelial cells infected with IAV showed that the basal cells are more resistant to other cell subtypes in the viral infection [5]. This higher resistance was associated with improved levels of IFITM3 and IFITM1 that are potent anti-IAV factors. On the other hand, respiratory epithelial cells infected with IAV produce several inflammatory cytokines that attract and activate immune cells. This cytokine-mediated recruitment and activation of immune cells can contribute to viral clearance or induce damage to the epithelial–endothelial barrier. Among these cytokines, it was shown that IAV infection induces the secretion of IL-6, IL-8, and granulocyte macrophage colony-stimulating factor (GM-CSF) by respiratory epithelial cells [29,30]. Of note, work comparing the cytokines induced by human primary respiratory epithelial cells infected with seasonal H1N1, 2009 pandemic H1N1, and highly pathogenic H5N1 IAV showed that both expression and protein levels of IL-8 were significantly higher in cells infected with the H5N1 subtype [31]. A study evaluating polymorphisms and the levels of inflammatory cytokines in patients infected with IAV and their association with disease severity and fatality found that IL-6 concentrations were significantly higher in fatal cases compared to those of survived severe cases [32]. Similarly, IL-6 levels were found to have a positive correlation with the severity of pneumonia in IAV-infected patients [33]. Experiments with human lung A549 cells showed that IAV stimulates the expression of the suppressor of cytokine signaling-3 conducting to altered NF-κB activation and overexpression of IL-6 [34]. In contrast, studies in IL-6^−/−^ mice showed that the deficiency of this cytokine induces lower macrophage recruitment and activation after the challenge with IAV, which correlated with higher body weight loss and lethality [35]. Thus, PBE cells could be used as an in vitro system to evaluate the positive and negative roles of IFNs, antiviral factors, and inflammatory cytokines in the porcine respiratory epithelium in response to SIV infection.

The modulation of immune factors by SIV and the different impact of the subtypes in such response observed in this study are in line with previous works evaluating the viral infection in porcine respiratory epithelial cells. The challenge of MK1-OSU cells significantly reduced the expressions of the viral PRRs TLR7 and MDA5 [14]. Interestingly, SIV H3N2 (A/swine/Iowa/0855/2007) induced higher decrements than the H1N1 (A/swine/Minnesota/2073/2008) subtype. The innate immune response triggered by SIV H3N2 (strain A/Swine/Bissendorf/IDT1864/2003) was evaluated in the NPTr cell line [18]. The infection increased IFN-β, IFN-λ1, Mx1, OAS1, PKR, and RIG-I but it did not alter IFN-α and IFN-λ3 expressions. In addition, increases in IL-6, IL-8, and SOCS1 were detected in NPTr cells infected with SIV H3N2 while IL-1β and TNF-α were not modified. Studies in siTEC and siNEC cells infected with four different SIV strains also detected virus subtype-specific changes in the modulation of IFNs and antiviral factors [36]. SIV infection upregulated the expressions of type I and III IFNs in both cell lines. However, tracheal cells were less efficient than nasal cells to express IFN-λ, which was associated with the fact that siTECs were more permissive than siNEC cells to viral replication. In addition, the upregulation of IFN-β, RIG-1, IRF7, and the guanylate binding protein 1 (GBP1) in nasal cells was higher for SIV H1N2 (A/swine/North Carolina/156551/2015) and H3N2 (A/swine/North Carolina/157674/2015) compared to H1N1 (A/swine/North Carolina/154072/2015).

The immune response is regulated by both stimulatory and inhibitory signals. Positive regulators are important to trigger the mechanisms that mediate pathogen clearance, while negative regulators modulate the inflammatory response to limit tissue damage without affecting pathogen elimination [37]. Since overactive immune response causing severe tissue damage is detrimental for the host, several negative regulators are modulated in the respiratory tract during the course of IAV infections including A20 [38], BCL-3 [39], and MKP-1 [40]. In line with these previous studies, we observed here that the infection of PBE cells with either H1N1 or H3N2 SIV increased A20, BCL-3, and MKP-1 expressions.

A20 (TNF alpha-induced protein 3 or TNFAIP3) is a cytoplasmic ubiquitin-editing protein that negatively regulates the activation of IRF3 mediated by TLR3 and RIG-I. Studies have reported that the non-structural protein NS1 of IAV significantly induces the protein levels of A20 in human lung A549 cells [41]. The induction of this negative regulator by IAV infection was corroborated in human bronchial epithelial cells and the lung from mice [42]. It was also shown that the overexpression of miR-29c in A549 cells prior to the IAV infection protects A20 transcripts conducting to decreased NF-κB activity and proinflammatory cytokines expression [43]. These studies show that the upregulation of A20 is associated with the modulation of NF-κB activation in respiratory epithelial cells and the protection against inflammatory injury in the context of IAV infection. Interestingly, A549 cells exposed to a chronic low dose of LPS exhibit increased A20 expression in response to IAV infection. This increase enhances peroxisome proliferator-activated receptor (PPAR)-α and PPAR-γ, inhibiting NLRP3 inflammasome activation and NF-κB signaling [44]. The same work described that BALB/c mice exposed to a low dose of LPS prior to IAV H1N1 (A/Fort Monmouth/1/1947) infection exhibited reduced weight loss, lung injuries, and respiratory levels of TNF-α, IL-6, and IL-18. It was reported that mice with the specific deletion of A20 in airway epithelial cells (A20^AEC-KO^ animals) exhibited lower levels of IL-6, the murine IL-8 homologue KC, MCP-1, and neutrophils in the respiratory tract after the challenge with TNF-α or the TLR3 agonist poly(I:C) compared to control mice [38]. Surprisingly, A20^AEC-KO^ mice were better protected against H3N2-mouse-adapted IAV than wild-type controls, an effect that was associated with reduced MCP-1 production in the lungs and lower inflammatory damage. This work highlighted that the role of A20 in the resistance to IAV infection is not completely understood and that further research is needed to characterize this negative regulator in the context of the antiviral immune response. On the other hand, BCL-3 is an oncoprotein that can inhibit the expression of inflammatory factors. It is a member of the IκB family, which acts as an inhibitor of NF-κB [45]. Although it was reported that BCL-3 deficiency affects T cell-dependent immunity against IAV [39], the role of this protein on the anti-influenza innate immune response triggered by respiratory epithelial cells has not been investigated before. Interestingly, it was reported that respiratory syncytial virus (RSV) infection induces BCL-3 expression in A549 cells inhibiting IL-8 production [46]. The BCL-3 protein induction in RSV-infected airway epithelial cells has then been associated with the termination of chemokine expression to control inflammation. [47]. MKP-1 is also a negative regulator of inflammation through its action in the MAPK pathway. Comparative studies using Mkp-1^+/+^ and Mkp-1^−/−^ mice showed differences in the response to IAV infection in terms of viral replication, host physiology, and immune response [40]. It was described that p38 and JNK MAPKs activation was prolonged in the respiratory tract of Mkp-1^−/−^ mice and transcriptomic studies showed higher expressions of inflammatory factors in these animals compared to Mkp-1^+/+^ controls upon IAV infection. The higher inflammatory response correlated with accelerated weight loss and hypothermia.

Of note, when the expressions of SIGIRR and Tollip were analyzed in PBE cells after SIV infections, it was observed that only the cells challenged with the subtype H1N1 upregulated these regulatory factors. SIGIRR can prevent the recruitment of the adaptor protein MyD88 during the activation of TLRs, thereby inhibiting downstream inflammatory pathways [48], while Tollip is an adaptor protein expressed in epithelial cells and macrophages, involved in regulating TLR- and IL-1-mediated signaling pathways [49]. Both regulators have been associated with the modulation of inflammatory and antiviral responses in the respiratory tract. It was shown that human tracheobronchial epithelial cells isolated from donors possessing the Tollip single-nucleotide polymorphism rs5743899 and in vitro-challenged with rhinovirus expressed fewer anti-viral genes and produced more IL-8 compared with cells from different genotypes [50]. The role of Tollip in the regulation of neutrophilic inflammation and the protection of lungs during rhinovirus infection was also confirmed in vivo in a mice model [51]. Furthermore, studies performed in wild-type and Tollip^−/−^ mice showed that the infection with IAV was more severe in the latter group [52]. Mice deficient in Tollip had significantly higher IAV loads, body weight loss, IL-33 release, and neutrophilic inflammation in lungs. More recently, it was demonstrated that Tollip cooperates with the surfactant protein A secreted by lung alveolar type II cells to inhibit IAV in mice [52] and that the exposure of the respiratory epithelium to cigarette smoke reduces Tollip expression via epigenetic mechanisms, increasing the susceptibility to IAV infection [53]. On the other hand, SIGIRR was shown to modulate the production of IL-6 and TNF-α in human airway epithelial cells induced by the activation of the antiviral innate sensor TLR9 [54]. Reduced expression of this regulator has been also described in airway epithelial cells from cystic fibrosis patients and this SIGIRR reduction was associated with deficient anti-viral activity mediated by TLR3 conducting to increased viral replication and dysregulated proinflammatory cytokine production after rhinovirus or IAV infections [55]. Considering these previous studies that demonstrated the protective role of Tollip and SIGIRR in the context of respiratory viral infections, the fact that H1N1 induces the expressions of both regulators, and H3N2 not, could be associated with the lower cytopathic effect induced in PBE cells by the first SIV subtype, particularly in the alteration of cilia. Several transcription factors have been shown to modulate the expression of genes involved in cilia assembly and function including RFX, FOXJ1, and TAp73 [56]. Although respiratory pathogens like IAV can induce direct detrimental effects on cilia function and expression, it was demonstrated that the inflammatory response to infection can also alter respiratory cilia function and development [57]. The role of Tollip and SIGIRR as well as other negative regulators of the TLR signaling in the generation of antiviral responses and the resistance to respiratory viruses in the porcine respiratory epithelium has not been evaluated before. The PBE cell line can be a useful in vitro tool to investigate this topic.

## 4. Materials and Methods

### 4.1. Cell Culture and Treatments

The PBE cell line used in this study was derived from the bronchial tissue of a 7-day-old piglet as described before [17]. The primary cultures of respiratory epithelial cells were cloned using the limiting dilution method after several passages and transfected to ultimately obtain the PBE immortalized cell line. PBE cells were cultured in DMEM (GIBCO, Grand Island, NY, USA), with 10% FCS and 1% penicillin/streptomycin at 37 °C with 5% CO_2_ in a humidified atmosphere (Thermo Fisher Scientific, Waltham, MA, USA; CO_2_ incubator).

PBE cells were infected with SIV at MOIs of 0.01 or 0.001 supplemented with 1 μg/mL TPCK-trypsin for 1 h. Mock-treated cells (controls) received virus-free culture medium supplemented with 1 μg/mL TPCK-trypsin for 1 h. After infection, the culture medium was discarded, and the PBE cells were subsequently washed twice with prewarmed (37 °C) culture medium and then cultured with TPCK-containing medium. PBE cells were harvested for RNA isolation at 3, 6, 12, 24, 36, and 48 h. All experiments were performed in triplicate. For virus titration assays, the supernatants were harvested at 1, 2, 3, 4, and 5 days. The supernatants were collected by centrifugation (10,000× *g*, 15 min, 4 °C) and the 50% tissue culture infectious dose (TCID_50_) assay was used.

### 4.2. Virus and Titration

The SIV H3N2 subtype (A/swine/Kagoshima/37-7201/2019) and H1N1 subtype (A/swine/Tottori/41-6255/2018) were provided by the Division of Infectious Animal Disease Research, National Institute of Animal Health, National Agriculture and Food Research Organization (Tsukuba, Japan) and propagated in Madin–Darby canine kidney cells (MDCKs). Viral stocks were titrated on MDCK cells. Briefly, the virus was serially diluted with serum-free DMEM (GIBCO, NY, USA) supplemented with 1 μg/mL tosylsulfonyl phenylalanyl chloromethyl ketone (TPCK)-trypsin (Sigma, St Louis, MO, USA) and 100 μL of the sample were inoculated onto a monolayer of MDCK cells in a 96-well plate and incubated for 168 h. Then, the TCID_50_ assay was performed using the Reed–Muench method.

### 4.3. Immunofluorescence Staining for SIV

The PBE cells were seeded on a collagen-coated 96-well culture-slide at a cell density of 1 × 10^4^ cells/cm^2^ for 3 days, washed with cold PBS once, and then fixed with 70% acetone for 5 min at −4 °C. PBE cells were blocked with 1% goat serum in PBS for 1 h and incubated with primary antibody (Influenza A virus Nucleoprotein antibody [HL1078], Gene Tex, Irvine, CA, USA) diluted at 1/500 in 1% goat serum for 60 min at 25 °C. Cells were washed five times and incubated with the secondary antibody (secondary Alexa Fluor 488 conjugated Goat anti-rabbit IgG antibody, A-11008, Thermo Fisher Scientific, MA, USA) diluted at 1:2000 in 4% goat serum for 1 h at room temperature. Then, cells were treated with 40,6-diamidino-2-phenylindole (DAPI) for 10 min at room temperature in the dark and washed three times with PBS. Non-infected PBE cells treated with both primary and secondary antibodies were used as controls. A BZ-9000 laser scanning microscope (Keyence, Tokyo, Japan) was used to analyze the samples. PBE cells were photographed at 200× with the BZ II Viewer software, version 1.4.0.0.

### 4.4. Transepithelial Electrical Resistance (TEER)

PBE cells were seeded on a collagen-coated inserts 24-well plate (0.4 μm pore size, 354444, Corning, AZ, USA) at an initial concentration of 1 × 10^5^ cells/well. TEER was measured using an epithelial volt–ohm meter with a chopstick electrode (Millicell ERS-2, MERS00002, EMD Millipore, Billerica, MA, USA). Triplicate measurements were recorded for each monolayer. The cells treated only with serum-free culture medium were used as a blank TEER value. Final TEER after blank subtraction was expressed as ΔTEER Ω/cm^2^.

### 4.5. Real-Time Quantitative PCR

Total RNA was extracted from PBE cells harvested at 0, 3, 6, 12, 24, 36, and 48 h after SIV challenge using TRIZOL reagent (Invitrogen, Carlsbad, CA, USA) according to the manufacturer’s protocols. RNA was quantified by measuring absorbance at 260 nm (NanoDrop^®^ ND-1000 Spectrophotometer, Thermo Fisher Scientific, MA, USA). The ratios of absorption (260/280 nm) of all samples were between 1.8 and 2.0. Aliquots of RNA were subjected to electrophoresis through a 1.4% agarose formaldehyde gel to verify their identities. Reverse transcription was performed with the Prime Script RT reagent Kit (Takara Bio, Shiga, Japan) following the manufacturer’s instructions. The quantitative real-time PCR was conducted on a CFX Connect Real-time PCR System (Bio-rad, Hercules, CA, USA) using TB Green Premix Ex Taq (Takara Bio, Shiga, Japan) according to the manufacturer’s recommendations. The thermal cycling conditions were 95 °C for 30 followed by 40 cycles at 95 °C for 5 s and 60 °C for 30 s. The primers used are listed in Supplementary Table S1. The study included IFNs, antiviral factors, inflammatory cytokines, and the regulators BCL-3, A20, MKP-1, SIGIRR, and Tollip.

The stable expression of β-actin across various pig tissues is used as a housekeeping gene, according to our previous publication [17]. A calibration curve derived from serially diluted plasmids was used to calculate the mRNA expression level. The mRNA expression level in each sample was normalized to the β-actin expression and then expressed as relative with the control set as 1.

### 4.6. Scanning Electron Microscopy (SEM)

Scanning electron microscopy was used to observe the morphological cytopathogenicity induced by SIV H1N1 or H3N2 on PBE cells. The cells were seeded at a concentration of 10^5^ cells per well and cultured on polyethyleneimine-coated cover slips (10 mm diameter) in 24-well plastic tissue culture plates. The viral infection was performed as described before. Then, the PBE cells were prepared according to the established protocol for SEM observation. Briefly, after washing twice with PBS buffer, the cells were fixed for 1 h at room temperature with a solution of 2.5% glutaraldehyde and 2.0% paraformaldehyde in 0.1 M cacodylate buffer (pH 7.4). Then, cells were washed, dehydrated in ethanol, treated with isoamyl acetate, and dried to a critical point with HCP-2. The cells were mounted on stubs, coated with gold, and observed with a scanning electron microscope (JSM5310/LV, JEOL Company, Tokyo, Japan).

### 4.7. Statistical Analysis

All results are shown as the mean and standard deviation from at least three independent experiments. Statistical analyses were performed with one-way ANOVA. *p*-values < 0.05 were considered statistically significant.

## 5. Conclusions

Immortalized respiratory cell lines from pigs can be useful in vitro systems for the study of viral infections and immune responses. These studies are of importance in the context of IAV infections not only for the agricultural field because pigs are natural hosts of these viruses but also because these animals serve as intermediate reservoirs of viruses that can threaten humans’ health. We previously developed the PBE cell line that was shown to trigger a complex antiviral innate immune response after the activation of the TLR3 signaling pathway. In this work, we advance in the characterization of this cell line by demonstrating that SIV can infect PBE cells inducing cytopathic effects and triggering the expression of IFNs, antiviral factors, inflammatory cytokines, and negative regulators of the TLR signaling that have been shown to play important roles in the outcomes of IAV infections in humans, pigs, and mice models.

## Data Availability

The original contributions presented in this study are included in the article. Further inquiries can be directed to the corresponding author(s).

## Supplementary Materials

The following supporting information can be downloaded at https://www.mdpi.com/article/10.3390/ijms26125764/s1.
