# Supplementary material for: The Originally Established PBE Cell Line as a Reliable In Vitro Model for Investigating SIV Infection and Immunity
Source: Int J Mol Sci. 2025 Jun 16;26(12):5764. doi: 10.3390/ijms26125764 (PMC12193638; doi:10.3390/ijms26125764)
Supplement: Supplementary file 1 [file ijms-26-05764-s001.zip › ijms-3683618-supplementary.pdf]

Supplementary Table S1. Sequences of primers used for PCR.

β-actin	Forward	CAT CAC CAT CGG CAA CGA
	Reverse	GCG TAG AGG TCC TTC CTG ATG T
IFN-β	Forward	AGT TGC CTG GGA CTC CTC AA
	Reverse	CCT CAG GGA CCT CAA AGT TCA T
Mx1	Forward	GAG GTG GAC CCC GAA GGA
	Reverse	CAC CAG ATC CGG CTT CGT
Mx2	Forward	AATCATCACCAGGTGTCCGC
	Reverse	CTTTGCGTATTTCCCGCTCC
OAS1	Forward	GAG CTG CAG CGA GAC TTC CT
	Reverse	TGC TTG ACA AGG CGG ATG A
OAS2	Forward	CAG CCA GAG CAA TGG GAA AC
	Reverse	CCT CTG GCC ACG CTT ATC AC
OASL	Forward	TTC AAG AAG TGT GCG TGT GC
	Reverse	TGT CTC AAG AGC ACC GCT TT
IFITM1	Forward	ATT TGT TCC ACC CTC CCA GG
	Reverse	TTG ATG CAG AGA CGG AGC AG
SIV NP	Forward	AAG CAG GGTAGA TAA TCA CTC
	Reverse	GAG CAC CAT TCT CTC TAT TGT TA
BCL-3	Forward	GCTAGGATCCATGGCCACCCGTGCAGATGAGGAC
	Reverse	GCTAAAGCTTCAGCTGCCTCCTGGAGCTGGGGAGGG
A20	Forward	TAGCGGCCGCTAGTGTCATCATGGTGCTATCCTCTG
	Reverse	CAGAGGATAGCACCATGATGACACTAGCGGCCGCTA
SIGIRR	Forward	CCT CCT TCA CTC TTC AGA GAG C
	Reverse	ACG GCA CTT GAC ATA GAG CAG G
MKP-1	Forward	AGA TCC TGT CCT TCC TGT ACC
	Reverse	AGT CAA TAG CCT CGT TGA ACC
Tollip	Forward	GCG AAT TCG GAT GGG CGA CCA CCG TCA GCA C
	Reverse	GCG GTA CCC TAG GAC TCC TCG CCC ATC TGG
IL-6	Forward	GGA GAC TTG CCT GGT GAA AA
	Reverse	GTC AGG GGT GGT TAT TGC AT
IL-8	Forward	TTG GCA GCC TTC CTG ATT TC
	Reverse	AAC TTC TCC ACA ACC CTC TGC A
